# 1795. Evaluation of Appropriate Daptomycin Usage in an Academic Medical Center

**DOI:** 10.1093/ofid/ofac492.1425

**Published:** 2022-12-15

**Authors:** Nour A Daoud, Mollie VanNatta, Satya Koppada, Mohammad Alam, Muhammad Khan

**Affiliations:** LSU Health Science Center Shreveport, Shreveport, Louisiana; Ochsner LSU Health Shreveport, Shreveport, Louisiana; LSU Health Science Center Shreveport, Shreveport, Louisiana; Louisiana State University Health Sciences Center, Shreveport, LA, USA, shreveport, Louisiana; LSU Health Science Center Shreveport, Shreveport, Louisiana

## Abstract

**Background:**

The usage of daptomycin at our institution has steadily increased, with a threefold increase from Jan 2020 to Jan 2022 according to Days of Therapy Data. The increased usage prompted this study to determine whether daptomycin use at our institution is appropriately indicated and supervised by the infection disease service.

**Methods:**

We conducted a retrospective descriptive study on 74 patients who were selected randomly from a total of 234 pts who received daptomycin at our facility in Northwest Louisiana over the past two years. We studied the patient's age and gender, type of infection, indications, empiric versus targeted therapy, culture results, infectious disease consultation, and reason for early discontinuation.

**Results:**

Seventy-four pts were included in the analysis. The median age was 53.50 (22-90 yrs), and 47 pts (63.5%) were male. The most common infections were bone and joint (37, 50.7%), followed by bacteremia (22, 30.1%), followed by infective endocarditis (4, 5.5%). The infectious disease service was consulted before administration of daptomycin in 87.3% (62 patients). In 70.3% (52 patients), daptomycin was used as targeted therapy. MRSA was the most common bacteria for which daptomycin was used (29, 42.6%), followed by Enterococcus faecalis (10, 14.7%) and Coagulase-negative staphylococcus (8, 11.8%). Fifty-five pts (74.3%) received vancomycin before switching to daptomycin. The most common indications was MRSA with vancomycin MIC ≥ 2 mcg/ml (25, 33.8%), followed by as an alternative to vancomycin in acute renal failure (14, 18.9%), followed by clinical or/and microbiological failure on the previous antibiotic regimen (10, 13.5%). Thirty-six pts (48.6%) completed their course, 20 (27%) pts had early discontinuation, 11 pts (14.9%) were lost to follow-up, 4 pts (5.4%) were still on daptomycin at the time of data collection, and 3 pts (4.1%) died during hospitalization. The most common cause of early discontinuation was the culture growing pathogen necessitating discontinuation of daptomycin (7 patients, 35%), followed by insurance issues that account for 15%.

**Conclusion:**

Most daptomycin usage in our institution was wisely indicated under the supervision of the infectious disease service and was primarily used as targeted therapy.

**Disclosures:**

**All Authors**: No reported disclosures.

